# Antecedents of the effectiveness of entrepreneurship policy: An integrated framework

**DOI:** 10.1371/journal.pone.0247988

**Published:** 2026-02-11

**Authors:** Mingyang Zhang, Wei Zhang

**Affiliations:** 1 School of Public Administration, Central China Normal University, Wuhan, Hubei, P.R. China; 2 School of Public Administration, Sichuan University, Chengdu, Sichuan, P.R. China; China University of Mining and Technology, CHINA

## Abstract

By employing grounded theory method to conduct in-depth interviews, this paper argues that policy formulation, scientific and technology support, legal safeguards, policy implementation, and social environment are the major factors to influence the effectiveness of entrepreneurship policy of government, and thus comprehensively form an “effectiveness-testing” model to further analyze the pathway and internal mechanism. The findings show that these factors have an important effect on the effectiveness of entrepreneurship policy of government together with different functions. The findings are of great theoretical and managerial implications for perfecting and developing entrepreneurial management theory, public policy evaluation, and entrepreneurship policy management, as well as promoting local entrepreneurship and innovation activities.

## Introduction

Entrepreneurship plays a crucial role in creating innovation, improving competitiveness, generating new ideas, creating jobs, and promoting social adjustment and economic growth [1] in both developed and developing countries. In recent years, China has attached more and more importance to the role of innovation and entrepreneurship, and has made the innovation-driven strategy one of our basic national policies. The State Council of China has issued more than 60 important documents related to innovation and entrepreneurship, especially it issued “Opinions on policies and measures to vigorously promote mass entrepreneurship and innovation” in Guofa [2015] No.32 document on June 15, 2015 since Premier Li Keqiang put forward the concept of “mass entrepreneurship and innovation” in the government work report in 2015. Following this line of thought, innovation and entrepreneurship have reached unprecedented heights at national strategic level.

In fact, countries all over the world have established entrepreneurship policies with different priorities. Entrepreneurship policy refers to the focus of every economy, governments provides a variety of policy guarantees to promote entrepreneurial activities [2]. The existing research has mainly focused on three aspects. First, entrepreneurship policy has direct and indirect influence on entrepreneurship. Lundstrom & Steveson [3], Verheul, Wennekers, Audretsch, & Thurik [4], Smith [5], Sternberg [6]^‌‌^, and Kayne [7] classify entrepreneurship policy from diverse angles for different research purposes, and reveal how those factors influence entrepreneurial activities and entrepreneurship in the macro development environment. Although there is no consensus on the most effective policy tools, an analysis of policy affecting entrepreneurship is necessary. Specifically, for young innovative companies, government-guaranteed bank loan programs and venture capital equity investment financial incentives are promoting different roles [8]. Although business regulations may have a negative impact on entrepreneurial activities, the location of the policy does not show any measurable impact [9]. In other words, it is necessary to implement entrepreneurship policy to create entrepreneurial economy and realize economic benefits. Aiming at the relationship between entrepreneurship and the economy, Figueroa-Armijos & Johnson [10] used a quasi-market framework to analyze the impact of entrepreneurship policy on Kansas’ economy. In addition, researches show that the implementation of intellectual property legislation [11] and women’s entrepreneurship policy [12] have positive impacts on entrepreneurship. These studies have provided help to improve the level of entrepreneurship. However, these fragmented policies appear weak in terms of greatly improving the level of entrepreneurship.

Second, some scholars start the study on the theoretical framework of the mechanism of entrepreneurship policy. The collective framework of Lundstrom et al. [3]^‌‌^ has been widely accepted by scholars and has made pioneering contributions to the interpretation and evaluation of entrepreneurship polies by integrating six entrepreneurship policies (start-up fund support, entrepreneurship support, reducing barriers to entry, entrepreneurship promotion, entrepreneurship education, and controlling the number of target people) with incentives, skills, and opportunities. Tsai and Kuo [13] come up with the decision model of entrepreneurship policy by integrating the methods of DEMATEL, ANP, and ZOGP. In addition, O’Connor [14] proposed the purpose of entrepreneurship education from the perspective of economic theory, and established a policy framework supported by the policy background of the Australian government. These basic analytical frameworks help to understand entrepreneurship policy. In terms of policy diffusion, Norback, Persson, & Douhan [15] studied entrepreneurship policy in the form of entry costs in a lobbying model, explaining the trends in support of entrepreneurship policy around the world. In terms of policy design, Audretsch [16] emphasized to pay attention to all aspects of entrepreneurship. These analytical frameworks provide a solid foundation for the formulation and evaluation of entrepreneurship policy. However, the downside is that the antecedents affecting entrepreneurship policy are still unclear.

Third, some scholars have paid attention to the measurement and evaluation of entrepreneurship policy to further study its effectiveness. Tsai et al. [13] construct a comprehensive evaluation model of entrepreneurship strategy by taking the practice of entrepreneurship policy in Taiwan as an example to help policymakers for decision-making analysis in line with interdependent criteria and limited resources. Rigby & Ramlogan [17] find evaluation indicators to study additionality and net effect and determine the effectiveness and impact of policy by means of causal inference methods. Arshed, Carter, & Mason [18] explore and evaluate entrepreneurship policy in Britain from 2009 to 2010 and find that the reason why the policy is bad, because it is closely connected to the interests of policymakers. In terms of evaluation methods, Lundström et al. [19] introduced a method for evaluating SME taxpayers’ expenses and entrepreneurship policy. In addition, in order to improve effectiveness, Amorós, Poblete, & Mandakovic [20] believe that it is necessary to strengthen infrastructure (such as infrastructure, skills, and innovation) to promote the R&D and innovation mechanisms. These studies provide help in evaluating the effectiveness of entrepreneurship policy. However, there is still a lack of a multi-factor entrepreneurship policy evaluation model.

Drawing on the relevant extant literature, scholars pay more attention to the comprehensive effect of specific factors on entrepreneurial activities and entrepreneurs (entrepreneurial characteristics, entrepreneurial cognition), but they seldom analyze the intrinsic relationship between various environmental factors, such as leading role of policy on other factors. Although it has been the focus of academic attention to study how to determine the influence mechanism of environmental factors on entrepreneurial activities and how to measure the complex environmental factors, the researches on the evaluation and measurement of entrepreneurship policy is not enough, especially in lack of mature evaluation system and measurement method. More importantly, it has not yet identified factors affecting the effectiveness of entrepreneurship policy of government, so that it needs to be further explored by scholars from a macro policy perspective.

Therefore, based on the existing research results, this paper attempts to build an “effectiveness-testing model” to explore the factors influencing the effectiveness and its internal mechanism of entrepreneurship policy of governments. In this sense, this study is expected to provide theoretical and experiential reference for the government to formulate entrepreneurship policy, and thus, make contributions to social and economic prosperity.

This paper is organized as follows: In the next section, this paper first reviews the literature related to entrepreneurship policy and defines its effectiveness. Subsequently, the research method adopted in our study is presented. Next, it analyzes the data step by step by using grounded theory method. Thereafter, it outlines the theoretical framework indicating the factors that impact the effectiveness of entrepreneurship policy and reveals its mechanism in the model. Finally, this paper concludes with an account of theoretical contribution and managerial implications of this study.

## Entrepreneurship policy and its effectiveness

The scholars began to pay attention to entrepreneurship policy in academic circles since the end of the 19th century. However, scholars have not made systematic study on it until the beginning of this century, and there are few scholars and achievements specific to systematic study of it. Stevenson & Lundstrom [21] are the first define entrepreneurship policy and emphasize its importance. They believe that it is to take policy incentives as the start-up stage of the whole entrepreneurial process and achieve the goal of encouraging and stimulating entrepreneurship by solving the problems of design, incentives, opportunities, and skills. Gilbert, Audretsch, & McDougall [22] stress that public policy and governance could shape virtually all contextual determinants for the demand of entrepreneurship, and entrepreneurship policy often aims to catalyze better management, for instance, by promoting networks of potential customers and service providers, the uncertainty faced by new entrepreneurs is reduced. Mas-Tur & Simón-Moya [23] believe that the key to entrepreneurship policy lies in fund support for start-ups, because it is difficult for start-ups to get public assistance and subsidies compared with large enterprises.

Entrepreneurship policy covers a wide range of areas, from local to central levels of government, from low-tech to high-tech economic activities at the technical level, and it also consists of governance capacity, regulation, and poverty alleviation [22]. Entrepreneurship policy generally supports individuals interested in starting businesses and start-up businesses (activities of the previous three years) [19]. Based on the above research results, this paper defines entrepreneurship policy in the way that the government implements a series of policies in terms of capital, technology, taxation, and public services to improve the overall entrepreneurial level of the society, promote employment and stimulate economic growth, and so as to help establish new enterprises and develop entrepreneurial activities.

When researching policy, it usually involves the antecedents of policy and how to improve policy and policy processes, that is, policy theory and policy analysis [24]. Policy theory focuses on how explanatory variables form the policy process, and policy analysis tends to evaluate the effectiveness of policy [25]. The effectiveness of policy is often classified into the category of policy analysis. The impact of any specific policy is deep and extensive, because policy is often embedded in specific policy networks, structures, and social contexts and play a key role [26]. Therefore, policy evaluation often needs to balance the policy objectives, policy priorities and commitments. However, policy objectives are often vague, and policy priorities and commitments often change. This brings difficulties to policy evaluation [27]. Policy evaluation makes a better sense for the government to resolve the issues of policy shortcomings and implementation, in a way to provide the basis for policy improvement and improve scientific and rational nature of government policy, by reflecting policy effectiveness. Salamon, Sokolowski & List [28] divide policy evaluation criteria into legitimacy, feasibility, efficiency, and fairness, and further summarize the viewpoints of previous scholars. Therefore, this paper defines policy effectiveness as taking reasonable measures to respond to social demands and achieve established policy objectives on the basis of fairness.

Entrepreneurship policy belongs to the cross-category of entrepreneurship management and policy evaluation. As both of them are characterized by broad scope and responsibility, it is difficult to define the effectiveness of entrepreneurship policy, neither does in relevant research literature. In this sense, this paper combines both characteristics of entrepreneurship policy and policy effectiveness, and defines the effectiveness of entrepreneurship policy as the effect attained by the government in implementing entrepreneurship policy, that is, the role of entrepreneurship policy in stimulating entrepreneurship, promoting innovation, and improving productivity, as well as the ratio of its output to the total economy.

## Research design and data source

### Research method

This research was designed on the basis of the method of grounded theory. The grounded theory is an “inductive method of theoretical development” proposed by Glaser & Strauss [29], which contributes to the formation of substantive or formal theory via heuristic abstract process [29]. Its purpose is to generate theory by systematically collecting and analyzing data, rather than testing preconceived ideas or hypotheses [30,31]. Despite it is a kind of qualitative research methods, it combines an in-depth and rich interpretation of qualitative research with logical and rigorous systematic analysis of quantitative research [32,29]. After 50 years of development, it has become one of the most popular research designs in the world [33]. Data on grounded theory came from a variety of sources, including interviews, observations, government documents, videos, newspapers, letters, books. Grounded theory is derived from data and illustrated by examples of data characteristics. It is an adaptive theory that can help prevent opportunists from using it.

It needs to follow the following principles when using grounded theory method [34], First, data collection and analysis are interrelated. Researchers have to start analyzing the first set of data to find out clues once they get the first set of data, and then incorporate all seemingly relevant questions into the next group of interviews and observations. Second, concepts are the basic units of analysis. Researchers use the conceptualized rather than the actual data, that is, original data. Third, categories are related. The concepts that refer to the same phenomena were grouped to form categories, not all concepts are categorized. The latter represents higher level and more abstract concepts than the former, and these categories were generated through a comparative analysis of similarities and differences. Fourth, it is theoretical sampling. The sampling of grounded theory is made on the basis of theories. Sampling is performed based on notions, attributes, dimensions, and changes rather than specific population samples, and time units. Fifth, it refers to continuous comparative analysis. Concepts arise from the continuous comparison of similarities and differences between events. In addition, as mentioned earlier, collaborative comparisons and grouping can help researchers prevent bias. Sixth, it takes patterns and changes into account. This means that researchers should take a close look at the regularity of data and which rules that are not obvious. Seventh, it develops and validates the hypotheses about the relationship between categories as much as possible in the research process. Only by following the above research principles can a complete theoretical model be constructed.

Birks & Mills [35] believe that grounded theory research has the following steps: initial coding and data classification; concurrent data generation, collection, and analysis; make memos; theoretical sampling; use of induction and outreach logic for continuous comparative analysis; continuous comparative analysis by using inductive and outreach logic; theoretical sensitivity; intermediate coding; core categories determined; advanced coding and theoretical integration. In short, the primary process is to carry out open coding, axial coding, and selective coding on the data collected, so as to extract the preliminary theoretical model. At the same time, theoretical saturation test is conducted, data is supplemented until the theory is saturated, and finally theoretical construction is completed [36].

**Fig 1:**
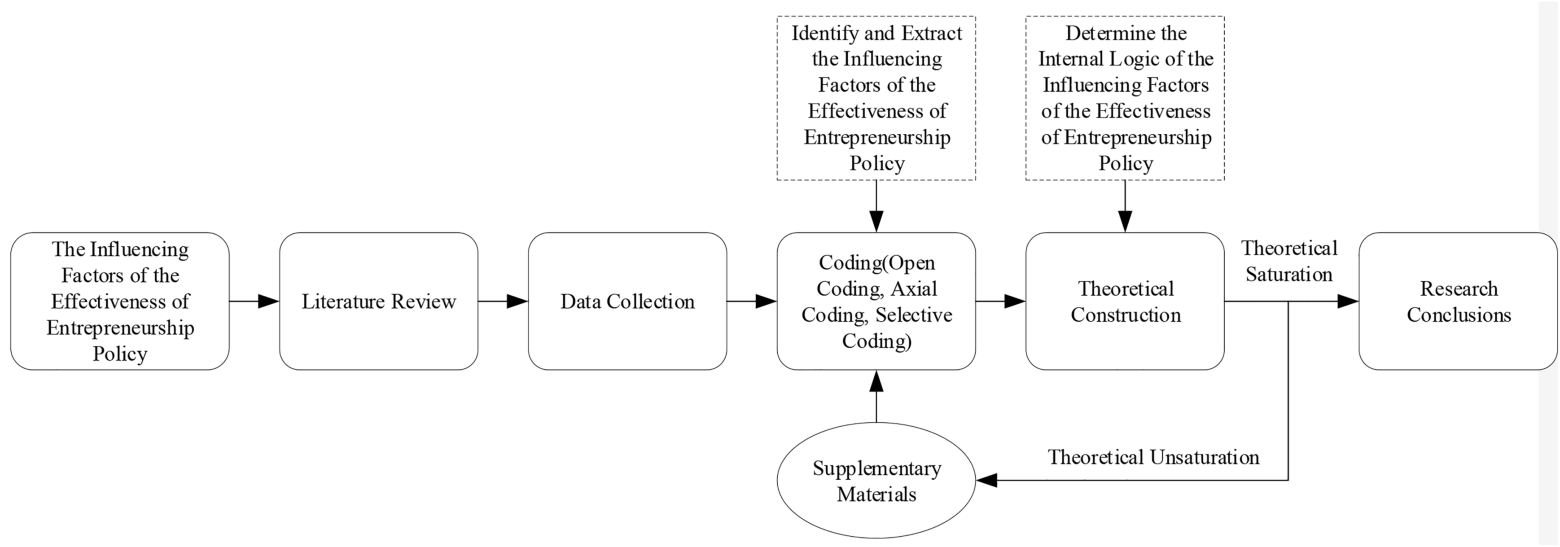
The logic of thinking in research.

This paper chooses grounded theory as the research method for the following reasons: First, most of the existing research on entrepreneurship policy is quantitative and lacks qualitative exploratory analysis. Second, many factors can affect the effectiveness of entrepreneurship policy. Considering the complexity of influencing factors, traditional hypothesis testing is difficult to perform [31]. Therefore, in this study, grounded theory can make up for the shortcomings of traditional quantitative research. Third, the relationship between the effectiveness of entrepreneurship policy and its influencing factors is dynamic and complex. Therefore, it is difficult for quantitative research to clearly explain the relationship between “how” and “why” between them. Because grounded theory is essentially explanatory, it aims to discover concepts and relationships and provide theoretical explanations for existing phenomena [30], thus in this research, grounded theory is advanced and useful. (See Fig 1).

### Data sources

This study was devoted to analyzing the influencing factors of the effectiveness of entrepreneurship policy, and data was collected from activities of entrepreneurial practice in Wuhan. The interviewed entrepreneurs came from Wuhan University, Central China Normal University, Wuhan University of Technology, Wuhan Hongshan District Venture Center, Optics Valley Venture Coffee, and Qingshan Mass Entrepreneurship Center. They also worked in different entrepreneurial fields, including information technology, intelligent manufacturing, energy conservation and environmental protection, Internet + , new energy, and traditional industries. The selected samples were representative enough to reflect the practical situation of innovation and entrepreneurship in Wuhan.

Considering the relationship between age and entrepreneurship, all the interviewees were in the age group of 20–40. People in this age group are the main force of entrepreneurship as they have strong entrepreneurial intention, strong adaptability, active thinking, and strong entrepreneurial ability. In accordance with the principle of theoretical saturation, the total number of samples was calculated until the interviewees could no longer provide important new information. Finally, 24 entrepreneurs were selected as interviewees, whose information statistics are shown in Table 1.

**Table 1:** Information statistics of interviewed entrepreneurs.

Total Number	24	Number	Percentage
Gender	Male	16	66.67%
	Female	8	33.33%
Age	20-25	4	16.67%
	26-30	8	33.33%
	31-35	7	29.17%
	36-40	5	20.83%
Entrepreneurial Field	Information Technology	4	16.67%
	Intelligent Manufacturing	4	16.67%
	Energy Conservation and Environmental Protection	4	16.67%
	Internet +	5	20.83%
	New Energy	4	16.67%
	Traditional Industry	3	12.50%
Education Level	Junior College Degree	4	16.67%
	Bachelor Degree	9	37.50%
	Postgraduate Degree and Above	11	45.83%

One week before the interview, the researchers made appointment with interviewees and introduced the research background, so that the interviewees could have a certain understanding of the subject of the interview and get ready for it. In addition, before the start of the interview, we obtained explicit written consent from each interviewee agreed us to use all the data and information of the interview. And these interviewees were told that their personal health data or identifying data would not be accessed during the entire interview. During the interview, flexible strategies were adopted according to gender, age and entrepreneurial field of interviewees, and in-depth interviews were conducted around three questions: (1) Do you know the current entrepreneurship policy issued by the government (school)? Is the government (school) propaganda in place? (2) What support and help the current government (school) provide to you in terms of entrepreneurship policy? In which ways can’t it help you? (3) What issues do you think affect the support and help from the entrepreneurship policy? What aspects of the current government entrepreneurship policy need to be improved? The above questions were layered in a progressive manner, which was conducive to guide the interviewees to put forward their true views. At the end of the interview, the information provided by the interviewees was summarized in time, and the researchers communicated with the interviewees again, so as to get feedback and further supplement and revise the information obtained.

## Data analysis

### Open coding

Open coding is to systematically sort out and analyze the data while collecting and arranging it, it needs to summarize word by word, refine, and encode the data line by line, thereby condensing and defining concepts and categories. In brief, this process is like the working principle of a funnel, which filters the originally broad and meaningless data into concepts and categories with practical significance and connection. In order to reduce the influence of personal bias on the results of the study, the original sentences of the interviewees were used as much as possible, and finally, a total of 660 original sentences were obtained. Two-thirds of the original sentences were randomly selected, and were summarized and extracted into 67 concepts and 26 categories as shown in Table 2 by carefully identifying and eliminating similar, cross-cutting, contradictory, invalid, and low frequent (less than three appearances) original sentences.

**Table 2:** Open coding.

No.	Categories	Original statements (initial concept)
1	Clear Objective	A09 Entrepreneurship policy should have a definite macro goal (Clarity) A12 Goals can be quantified, for instance, how many billion in benefits can be generated within a few years (Quantifiable) A21 I think every level of government should make specific policies and set their own goals (Policy Specific)
2	System Coordination	A02 There are many policies, but they are scattered (Scattered Policy) A06 Entrepreneurship policy intersects or conflicts with other policy (Incoordination) A15 It should be an organized and systematic policy system, and there are sub-policies, general policies and policies with different emphases (Systematic)
3	Content Perfection	A01 The policy should cover entrepreneurs in all industries (Policy objects) A06 The policy should give overall support to entrepreneurs (Overall Support) A12 Capital and financing are the keys to successful entrepreneurship; the government should focus on them (key support)
4	Dynamic Adjustment	A14 “The zombie policy” has a great impediment to the growth of enterprises (Importance of Policy Adjustment) A15 Policy should keep the pace of times, and achieve the balance of supply and demand (Adjustment on Demand) A18 I think the dynamic adjustment of policy cannot be too frequent, of course, it cannot be avoided (frequency of adjustment)
5	Scientific and Technological Innovation Ability	A04 Science and technology innovation ability is the key and core of our high-tech enterprises (Importance of Science and Technology Innovation Ability) A12 Here we can quickly learn about cutting-edge technology (Quick Access to Information) A15 High-tech can easily be converted into capital here (Technology transformation)
6	Research Infrastructure	A06 If there is no infrastructure, where will high-tech come from? (Importance of Infrastructure) A15 It’s best to have public and accessible experimental facilities (Public Openness) A17 The cost of laboratory facilities and equipment is very high, and it is a big burden (Experimental Cost)
7	Research Institutions	A05 The scientific and technological strength of a place depends on the number of universities and scientific research institutions (The Number of Research Institutions) A10 The level of scientific research institutions is very important, and the national level is better than the following level (The level of Institutions) A22 We need to see how much output “they” produce each year and how much can be applied (Research Results)
8	Funds Input	A06 It is important that the government spends more money on scientific research every year (Increasing funding) A08 The investment of scientific research expenditure as a percentage of GDP should be increased annually (Investment to GDP ratio) A13 Starting a business with insufficient funds, it is just like making bricks without straw (Importance of funds)
9	Intellectual Property Protection	A03 To protect intellectual property by the government is to protect talents (The role of intellectual property) A12 At present, the legal protection of intellectual property rights is not comprehensive enough (Imperfect law) A18 Laws on entrepreneurship and protection of intellectual property are poorly publicized, we will lose a lot once in trouble (Law publicity)
10	Risk Prevention	A02 There is not yet a set of laws to prevent entrepreneurship risks (legislative defects) A04 New problems arise every day in the Internet economy, but legislation is made always when something goes wrong (Lagging legislation) A16 Policy should correspond to law and prevent trouble before it happens (Corresponding to policy and law)
11	Policy Propaganda	A12 Propaganda of entrepreneurship policy is not strong enough, so that many people do not understand it (Propaganda work) A14 The uniform “plagiarism” of media propaganda lacks in-depth and unique analysis (In-depth interpretation) A20 There is no official website for propagating entrepreneurship policy (Propaganda method)
12	Policy Implementation Supervision	A05 Many people use policies to access to funds in unreasonable ways, but no one cares (Lack of supervision) A13 The media only publicize news from the positive side, some bad aspects are not to be exposed (Weak media supervision) A23 Government has a lot of momentum and actions, but no one knows what the result will be (Lack of performance)
13	Service Level	A09 There are many departments in charge, and we need to get it stamped by each department and thus gain permission (Multi-leadership) A11 The procedures for obtaining government assistance are complex and cumbersome, and some conditions are difficult for our small company to meet (Complicated procedures) A14 The government likes to procrastinate again and again, no matter it does business or allocates funds (Inefficiency)
14	Political System	A03 Macroscopic system stability is very important and conducive to the development of entrepreneurial activities (Political system stability) A09 Chinese political system is conducive to the sustained and smooth implementation of entrepreneurship policy (Advantages of the political system)
15	Political Behavior	A07 Large enterprises are often involved in government politics and are easily supported, because their growth may have direct benefits for the government. However, this is not necessarily a good thing for small enterprises (Enterprises` political behavior affects entrepreneurship)
16	Political Network	A15 If enterprises can have good relations with the government and leaders, with this kind of network, they can reduce risks, obtain information and resources which are not easily accessible to the general enterprises, and increase the exposure in the media. In a word, they are easier to succeed than others (Enterprises` political networks influence entrepreneurship)
17	Economic Aggregate	A10 Regional economic aggregate has a great influence on entrepreneurship, and the more developed it is, the more entrepreneurs it attracts (economic aggregate attracts entrepreneurs) A12 The higher the GDP, the more the opportunities to start a business (Creating business opportunities)
18	Per capita income	A06 People with high income have money to consume (Per capita income drives consumption) A21 Income is very important. I have been working for a few years to accumulate capital and then start a business (Accumulate entrepreneurial capital)
19	Financial Revenue	A13 The increase in government revenue indicates that the economic situation is good, and it is good for entrepreneurship (Reflecting economic situation) A18 The government’s support for our enterprises comes from the government’s revenue, so it is possible for us to increase subsidies if the revenue increases (Government expenditure on entrepreneurship)
20	Industrial Structure	A09 The higher the degree of industrial specialization is, the easier it is for entrepreneurs to obtain resources (Industrial specialization) A24 The more the industries in a region, the more the information we can learn (Industry diversification)
21	Entrepreneurial Identification	A05 Everyone said that I had “courage” and did not understand why I did not do my job and ventured to start a business (Social understanding) A12 Many entrepreneurs feel socially inferior and unrecognized (social identification) A22 Parents at home are behind The Times and don’t agree to start a business (Family identification)
22	Entrepreneurial Spirit	A01 The high income of entrepreneurship is accompanied by high risk (Venturous spirit) A11 The environment is based on stability, and risk is easy to go wrong (Environmental tone) A20 Colleges and universities teach too little about entrepreneurship (Entrepreneurship education)
23	Entrepreneurial Atmosphere	A10 There are many entrepreneurs around us, so I also want to try. (Number of entrepreneurs) A16 It is to attract people`s attention by creating entrepreneurship publicity atmosphere (Creation of atmosphere) A18 Like-minded entrepreneurs share common values (Values of entrepreneurship)
24	Population Density	A12 The population of Wuhan exceeded ten million, so it brought us tremendous business opportunities (Population number) A15 With a high population density, there is the more potential market and entrepreneurial demand (Population density affects entrepreneurial demand)
25	Population Mobility	A09 There is a large flow of people in Wuhan. People come here either to work or to start a business (Population flow) A14 Wuhan is an open city to all and has few barriers to entry for migrants (Barriers to migration)
26	Human Resource Quality	A03 There are many colleges and college students, so we are easy to find high-level employees (Rich human resources) A13 At present, college students have strong abilities and high technical level, and are easy to adapt to work (Ability level)

Note. “A” means the original sentences of interviewees, and the number after “A” is the ordinal number of interviewees.

### Axial coding

Compared with the broad definition of category in open coding, the purpose of axial coding is to find the relationship between various categories in the process of open coding. Each time, the researcher conducted in-depth discussion and analysis on a category, and classified registered related categories. Then the researcher found out the specific relationship between them to determine the main category and corresponding category. Through in-depth analysis, each category was divided into five main categories according to its internal logic, and its corresponding category and category connotation are summarized as follows: (see Table 3)

**Table 3:** Axial coding.

Main categories		Corresponding categories	Connotation of category
Policy Formulation		Clear Objective	The clarity, quantification and refinement of entrepreneurship policy objectives affect the effectiveness of entrepreneurship policy
		System Coordination	The systematicness and coordination of entrepreneurship policy affect the effectiveness of entrepreneurship policy
		Content Perfection	The content perfection degree of entrepreneurship policy affects the effectiveness of entrepreneurship policy
		Dynamic Adjustment	The dynamic adjustment of entrepreneurship policy affects the effectiveness of entrepreneurship policy
Science and Technology Support		Scientific and Technological Innovation Ability	The level of the city’s scientific and technological innovation ability affects the technology and information acquired by entrepreneurial enterprises
		Research Infrastructure	Research infrastructure affects research and innovation of entrepreneurial enterprises
		Research Institutions	Research institutions affect entrepreneurial enterprises to obtain technical and information support
		Funds Input	Government research funding input affects the development of technology-based entrepreneurs
Legal Protection		Intellectual Property Protection	The soundness and publicity of intellectual property protection laws affect entrepreneurial activities
		Risk Prevention	The improvement of the risk prevention legal system affects entrepreneurial activities
Policy Implementation		Policy Propaganda	The propaganda methods, propaganda efforts and in-depth interpretation of entrepreneurship policy affect the effectiveness of entrepreneurship policy
		Policy Implementation Supervision	The supervision and performance of entrepreneurship policy affect the effectiveness of entrepreneurship policy implemented
		Service Level	The administrative efficiency and process of the government affect the effectiveness of entrepreneurship policy implemented.
Social Environment	Political Factors	Political System	The political system affects entrepreneurship policy and entrepreneurial activities
		Political Behavior	Enterprises` political behaviors affect entrepreneurial activities
		Political Network	Enterprises` political network affects entrepreneurial activities
	Economic Base	Economic Aggregate	Economic aggregate affects entrepreneurial opportunities
		Per capita income	Per capita income affects market demand and entrepreneurial capital accumulation
		Financial Revenue	Financial revenue affects government investment in social entrepreneurship and entrepreneurs’ expectations.
		Industrial Structure	Industrial specialization and diversification affect entrepreneurial activities
	Cultural Atmosphere	Entrepreneurial Identification	Family’s and society’s understanding and identification of entrepreneurs affect their entrepreneurial intention
		Entrepreneurial Spirit	Entrepreneurial spirit affects entrepreneurial intention
		Entrepreneurial Atmosphere	Entrepreneurial atmosphere affects entrepreneurial intention
	Demographic Components	Population Density	Population density affects entrepreneurial opportunities
		Population Mobility	Population mobility affects entrepreneurial opportunities
		Human Resource Quality	The quantity and quality of human resources affect entrepreneurial activities

### Selective coding

**Fig 2:**
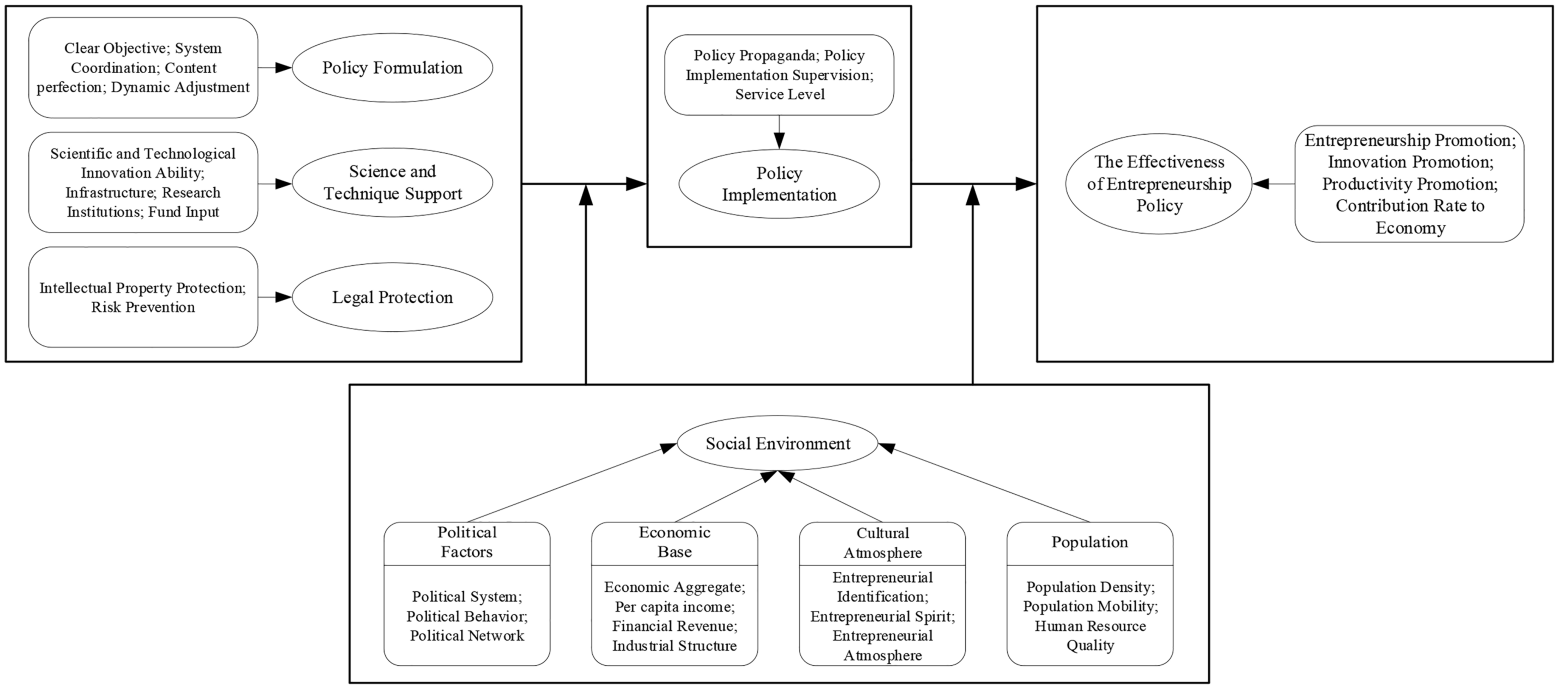
Model of influencing factors of the effectiveness of enterpreneurship policy.

Selective coding is to extract the core category according to the inherent logical relations between the main categories after the axis coding, and connect the core category with each main category in the form of “storyline”. The “storyline” plays an important role in this process and reveals the relational structure between the core category and the main categories. At the end of this process, the overall framework for model building of grounded theory was basically completed. After selective coding, this study indicated the core category of “the influencing factors and mechanism of the effectiveness of entrepreneurship policy”. Centering around this core category, four main categories were found to influence it, namely policy formulation, science and technology support, legal protection, policy implementation, and social environment. From this, it constructed the model of influencing factors for the effectiveness of entrepreneurship policy. (See Fig 2)

### Theoretical saturation test

After establishing the model of influencing factors for the effectiveness of entrepreneurship policy, the remaining one-third of original sentences was used for testing. Two findings are shown as follows: First, these categories formed by the remaining original sentences were basically contained in five categories: policy formulation, science and technique support, legal protection, policy implementation, and social environment, thus conforming to the internal logic of the model; second, the remaining original sentences have not formed new and important categories. Therefore, it can be concluded that the category mining in this model has been rich enough to reach theoretical saturation, which can be used to explain the factors influencing the effectiveness of entrepreneurship policy and its internal mechanism.

## Model interpretation and research findings

Through the research, it is found that the five categories of policy formulation, science and technique support, legal protection, policy implementation, and social environment have a significant impact on the effectiveness of entrepreneurship policy in the model. In this model, policy formulation, science and technique support, and legal protection directly determine whether the entrepreneurship policy is effective. They are in the position of independent variables in the model, emphasizing their importance. The conclusion is confirmed in Edelman & Yli-Renko`s [37] research. They believe that the emergence of new start-ups is attributed to the agreement of the resource characteristics of new entrepreneurs and the environment they are faced with, while policies, technologies and laws provide them with other resource attributes besides the industry background. Policy formulation, science and technique support, and legal protection can directly act on entrepreneurs` entrepreneurial intention. The above three factors improved can stimulate entrepreneurs’ desire to start businesses, thus promoting the prosperity of social entrepreneurship, driving employment, improving innovation, increasing social productivity and economic contribution. In this sense, the effectiveness of entrepreneurship policy can be reflected. Policy implementation plays an intermediary role, like a “bridge” in this model. Only through implementation can entrepreneurship policy have positive or negative effects on entrepreneurship. The study find that the way, strength, and problems of policy implementation have significant effect on the effectiveness of entrepreneurship policy. This conclusion responds to Ndemo’s [38] research result. He believes that the implementation process of entrepreneurship policy is very important. Corruption, bureaucracy, and inaction in this process have negative impact on the effectiveness of entrepreneurship policy so that policymakers should avoid these phenomena. Besides, in this model, the social environment was considered as the external environment for policy formulation and implementation to be able to affect the strength of the effectiveness of entrepreneurship policy. These external environments include political, economic, cultural, and demographic factors, which played a fundamental role in entrepreneurship policies and activities. These factors make the basic effect on entrepreneurship policy and entrepreneurial activities. This is consistent with Baker & Nelson’s [39] conclusion. They believe that the availability of external environment has an important impact on organizational behavior and results. Whether the external environment really affects the behavior of enterprises, the scarcity of resources and the usability patterns are all regarded as conditions for creating and affecting the birth and death of organizations. Due to the above factors affecting the development of entrepreneurial organizations, they have an indirect impact on policy formulation, implementation, and output. It will be explained in detail below.

### Policy formulation

Whether entrepreneurship policy is formulated successfully determines the effectiveness of entrepreneurship policy implemented fundamentally. Policy formulation includes four categories of clear objective, system coordination, content perfection, and dynamic adjustment. According to the interview content, first of all, from the perspective of entrepreneurs’ cognition, the government’s entrepreneurship policy does not have a clear overall goal, such as “*A09 entrepreneurship policy should have a definite macro goal*.” The ambiguity of policy objectives brings huge troubles to policy evaluation [27]. In the process of policy formulation, it is necessary to make clear what the overall goal is to achieve, how the target should be refined at all levels of governments, and whether it can be quantified and measured, which is of great importance to the effectiveness of entrepreneurship policy. On the contrary, governments at all levels do not know to what extent they want to achieve and only focus on blind action, resulting in inefficiency and poor output of entrepreneurship policy. Secondly, the macro-design of entrepreneurship policy is very important, and its systematicness and coordination affect the implementation effect of entrepreneurship policy. Whether policymakers have clear ideas determines whether their policies are organized. According to the interview, the current entrepreneurship policy is fragmented and uncoordinated. Specifically, it is manifested in the way that the government has many departments, each of them acts on its own and deals with businesses but in a lack of overall planning and systematicness; there are some overlapping or incongruous phenomena between entrepreneurship policies and industrial policies, which will affect the efficiency of policy implementation and result in reduced effectiveness. Thirdly, the integrity of entrepreneurship policy has an impact on its effectiveness. Smith [5] believes that entrepreneurship policy should include reducing administrative and bureaucratic burden of starting companies, obtaining small loans and seed funds, providing information services on entrepreneurship, setting examples for entrepreneurs, entrepreneurship education, promoting online services, incubators and guidance, and research and development tax incentives. To sum up, entrepreneurship policy should be composed of seven aspects: startup capital, financing, information service, infrastructure, entrepreneurship education guidance, taxation, and administrative burden. It is learnt that the current entrepreneurship policy basically covers the above seven aspects, but the policy demand is not balanced according to the interview. Among them, what entrepreneurs need most at the current stage are startup capital and financing policy, and this directly determines whether a startup can survive.

The study found that the entrepreneurship policy includes several aspects mentioned by Smith [5], while talent policy has a great influence on the effectiveness of entrepreneurship policy. In today’s knowledge-intensive business environment, enterprises must acquire and use a lot of knowledge to promote innovation and improve performance [40, 41]. Talent is an important carrier of tacit knowledge. Therefore, the talent policy is very important. Taking Wuhan as an example, the government of Wuhan has implemented a series of policies to attract high-level talents and college students to settle down and start businesses, which plays a positive role in the creation of the entire entrepreneurial atmosphere. However, this was rarely mentioned in previous studies. Finally, the dynamic adjustment of policy will also affect the effectiveness of policy. The policy is formulated in response to the social situation in a certain period. As time goes by, policies will not be suitable for the current situation. In this case, entrepreneurship policy will not produce positive effects but may have negative effects. As a result, it is necessary to adjust entrepreneurship policy accordingly. The government shall, according to the requirements of entrepreneurs, regularly adjust policy. Many interviewees mentioned this point, such as “*A15 policy should keep the pace of times, and achieve the balance of supply and demand*.” It thus can be found that entrepreneurs need the policy dynamically adjusted to promote the sustainable development of entrepreneurial activities. This has a continuing dynamic effect on the effectiveness of entrepreneurship policy.

### Science and technology support

There is a close relationship between scientific and technological progress and entrepreneurship, and technological development can be used as the driving force of entrepreneurship demand [42,4]. Science and technology support can improve entrepreneurs’ expectations of acquiring science and technology resources, enhance their entrepreneurial willingness and abilities, and thus have an impact on the effect of entrepreneurship policies. It includes scientific and technological innovation ability, infrastructure, research institutions, and fund input. Since no enterprise owns all technical resources internally [43], enterprises need to develop external connections to obtain diverse sources of knowledge and technologies outside their boundaries, thereby promoting the creation and combination of new technologies and knowledge [44]. In the interviews, it is found that entrepreneurs tend to be located in areas with high technological innovation capabilities, where they can obtain cutting-edge information and technology, this has a positive effect on technological innovation and development of entrepreneurial enterprises. Siqueira & Bruton [45] conduct an empirical study based on the resource dependence theory, and find that the level of technology is positively related to the company’s performance, especially for emerging economies. The result is similar to the conclusion of this study. Scientific research infrastructure and research institutions are the carriers of high technology, and the funds provide the fundamental support for them. Generally speaking, the higher the three indicators, the higher the ability of scientific and technological innovation, the more the high-tech achievements, and the greater the attraction to enterprises. The finding of Mosey, Guerrero, & Greenman [46] demonstrate that technology opportunities can be recognized and utilized by individuals through creating new enterprises. As a consequence, in regions with relatively high technology level, entrepreneurship can be driven by the transformation of achievements, and the positive effect it produces has a significant contribution to the output of entrepreneurship policy.

### Legal protection

According to the theory of knowledge spillover entrepreneurship, an environment with more knowledge will generate more entrepreneurial opportunities [47]. However, knowledge without legal protection will face huge risks. When the rights and interests of entrepreneurs and start-ups are effectively guaranteed, it is conducive to the success of entrepreneurship, thus increasing the output of entrepreneurship policy. In addition, when the phenomenon of successful entrepreneurship increases, it will affect potential entrepreneurs, attract them to enter the entrepreneurial field, create entrepreneurial atmosphere, and accordingly increase the output of entrepreneurship policy. Therefore, legal guarantee is an independent variable in this model, and its quality directly influences the implementation effect of entrepreneurial policy. The study found that entrepreneurs currently encounter two legal problems, the first is intellectual property right, the second is risk prevention. Firstly, due to the lack of legal propaganda of government and weak legal awareness of entrepreneurs, the problem of infringement is likely to occur to damage their legitimate interests. For example, “*A18 laws on entrepreneurship and protection of intellectual property are poorly publicized, we will lose a lot once in trouble*.” Moreover, the current law fails to adapt to the complexity of the definition and division of high-tech intellectual property, which makes the rights and interests of entrepreneurs vulnerable. From a macro perspective, the benefits of entrepreneurship policy are impaired. Secondly, the legal risk prevention of entrepreneurship is insufficient. emerging economies, especially those represented by “Internet +”, are new industries, but laws often lag behind. To some extent, it is difficult to effectively prevent new risks, resulting in market confusion. Entrepreneurship policy used by people may also foster “unhealthy tendency”, and the policy effect is difficult to achieve. Consequently, it is very important to overcome the lag of law, keep pace with the times, and establish a sound risk prevention system of entrepreneurship policy for the implementation effect of entrepreneurship policy.

### Policy implementation

Policy implementation consists of three categories of policy propaganda, policy supervision, and service level. First of all, entrepreneurship policy is to be understood through propaganda, so its propaganda intensity, propaganda methods and whether to carry on the thorough explanation to the policy appear particularly important. In the interview, we found that although the government was making great efforts to publicize, the publicity is still not strong enough. For example, “*A12 Propaganda of entrepreneurship policy is not strong enough, so that many people do not understand it*.” If people don’t understand the entrepreneurship policy, then the effectiveness of the policy will not be discussed. That is to say, only by making entrepreneurs and potential entrepreneurs understand that policy can help them succeed in starting a business can their willingness to start a business be enhanced. Following this line of thought, more efforts should be made in publicity, especially for college students. Only by making them understand the policy dividend, can government increase the actual audience of entrepreneurship policy, thus having a positive impact on the implementation of entrepreneurship policy. Besides, the interviewees reported more on propaganda channels and in-depth interpretation, for instance, “A20 there is no official website for propagating entrepreneurship policy” and “*A14 the uniform ‘plagiarism’ of media propaganda lacks in-depth and unique analysis*.” This will lead to two results: On the one hand, without a comprehensive inquiry system, it is difficult for entrepreneurs to inquire systematic and complete entrepreneurship policy. As mentioned above, this will make entrepreneurship policy invalid; on the other hand, publicity is superficial and formal, which makes it difficult for entrepreneurs to understand and make rational use of the policy and has a negative impact on the effectiveness of the entrepreneurship policy.

Secondly, policy supervision is an indispensable part of policy implementation and is directly related to whether the entrepreneurship policy can be implemented seriously. In the interview, it was found that the implementation of entrepreneurship policy was in lack of necessary supervision. For example, “*A05 many people use the policy to access to funds in unreasonable ways, but no one cares*.” This shows that there are loopholes and lax supervision in the implementation of policy, and the direct consequence is the waste and abuse of entrepreneurial resources. In other words, the government did not understand the effect of policy. Therefore, the lack of performance feedback of entrepreneurship policy makes it difficult to give feedback to policymakers about the implementation, which affects the dynamic adjustment of entrepreneurship policies and adversely affects their implementation effects.

Thirdly, the level of government services will affect the implementation of the entrepreneurship policy. In the interview, it was found that the one-stop service system still needs to be improved, the administrative procedures are complicated, and the entrepreneurs spend a higher cost on the administrative procedures. It will weaken the entrepreneurial intention and opportunities. The conclusion proves Smith`s [5] research results (reducing the administrative burden has a positive effect on entrepreneurship). The difference lies in that the multi-leadership and inefficiency of government departments will also cause adverse effects on policy implementation, thus affecting the effect of entrepreneurship policy.

### Social environment

Amara & Landry [48] emphasized that the introduction of highly innovative innovations requires broader sources of information and knowledge both inside and outside the organization. However, what they did not mention is that in the start-up stage of enterprises, entrepreneurs themselves are an important source of knowledge. Therefore, the pros and cons of the entrepreneurial environment will have an impact on whether entrepreneurs enter the entrepreneurial stage, thereby affecting the establishment of start-ups and the accumulation of knowledge. Social environment is an external influence factor of entrepreneurship policy. Through this study, it was found that these factors include political environment, economic base, cultural atmosphere, and demographic components. These factors have an impact on entrepreneurial opportunity, entrepreneurial costs, entrepreneurial expectation, and entrepreneurial intention, and they will strengthen or weaken social entrepreneurship activities. While social entrepreneurship is the carrier of entrepreneurship policy to be effective so that it indirectly affects the effectiveness of entrepreneurship policy. The social environment is the basis for the emergence and evolution of entrepreneurship policy, and it has an indirect effect on the effectiveness of entrepreneurship policy.

**Political factors.** Not only the stability of the team has an impact on enterprise performance [49], but the stability of the external political system is also particularly important. Baumol [50] emphasized the important role of the institutional environment in promoting and shaping entrepreneurship and innovation outcomes. Norms and institutions can guide entrepreneurship and thus constitute a key pillar of entrepreneurship and innovation policy. The findings indicate that political system, political behavior, and political network have impacts on the activities of entrepreneurs and the implementation of entrepreneurship policy. According to the interview content, it can be found that the stability of political system is also positively correlated with entrepreneurship. For example, it can provide continuous institutional guarantee and policy support for entrepreneurs. That affects the audience of entrepreneurship policy as well as the effectiveness of entrepreneurship policy. In addition, political behaviors and political networks of enterprises have an influence on entrepreneurship policy, which has not been mentioned before.

The political behaviors of enterprises are that enterprises influence the government behavior and obtain the resources their needs by participating in political activities. In the research, it is found that some large enterprises can influence the formulation and implementation of government policies. However, as entrepreneurial enterprises, they tend to be weak and have difficulty in obtaining “special” resources, so they are at a disadvantage in market competition, which is in line with the hypothesis of resource dependence theory [51]. At the same time, many interviewees mentioned in the interview that the political network of entrepreneurial enterprises plays a similar role above. By getting to know the key government figures, the exposure of enterprises and the possibility of obtaining resources can be increased [52]. For example, “*A15 if enterprises can have good relations with the government and leaders, with this kind of network, they can reduce risks, obtain information and resources which are not easily accessible to the general enterprises, and increase the exposure in the media. In a word, they are easier to succeed than others*.” Thus, it can be seen that the political behavior and political network of enterprises increase the uncertainty of the implementation of entrepreneurship policy, thus affecting its effectiveness. How to correctly guide the political behavior of enterprises and standardize the political network is also a question to be considered to improve the effectiveness of the entrepreneurship policy.

Economic base. Previous scholars have found that the implementation of entrepreneurship policy has a positive effect on improving the level of social entrepreneurship and economic development [53, 54], but less research has been reported on the impact of the economy on entrepreneurship. This study shows that the level of economic development can affect the effectiveness of entrepreneurship policy. First of all, the larger the regional economic aggregate is, the more developed the corresponding region will be, the greater the market demand will be created, and the more the entrepreneurial opportunities will be created. Based on this fact, entrepreneurship policy will produce more achievements. Secondly, the high per capita income can drive market demand and accumulate capital for starting a business. For instance, “*A21 income is very important. I have been working for a few years to accumulate capital and then start a business*.” The higher the regional financial revenue, the better the economic situation. This can provide both more budget for social entrepreneurship and an economic guarantee for entrepreneurship policy. Thirdly, in this study, the specialization and diversification of regional industrial structure can influence the effectiveness of entrepreneurship policy. The higher the degree of industrial specialization and diversification, the higher the quality and quantity of information and technology that can be provided to start-ups. The previous articles discussed the important influence of science and technology on the implementation of entrepreneurship policy, showing that industrial specialization and diversification can also bring positive impact on the implementation of entrepreneurship policy.

Cultural atmosphere. The entrepreneurial culture atmosphere is capable of exerting an influence on entrepreneurs’ entrepreneurial traits, entrepreneurial cognition, and entrepreneurial intention in a subtle way. By influencing the audience of entrepreneurship policy -- entrepreneurs, it can further indirectly affect the implementation effect of the policy. The study found that entrepreneurial culture atmosphere has the following three categories: entrepreneurial identification, entrepreneurial spirit, and entrepreneurial atmosphere. Entrepreneurial identification is reflected in whether entrepreneurs can be understood and recognized by their families and the society. The more social recognition the entrepreneurs gain, the more they can stimulate their entrepreneurial motivation to improve the vitality of social entrepreneurship and further affect the implementation effect of entrepreneurship policy. In entrepreneurial spirit, according to the interviews, entrepreneurship education is rare at present, and the social environment tends to be conservative to some extent, which is not conducive to the cultivation of entrepreneurship such as adventure and innovation. The regional entrepreneurial atmosphere is reflected by the number and common values of entrepreneurs, and the atmosphere of entrepreneurship propaganda. The stronger the entrepreneurial atmosphere is, the more favorable it will be to stimulate the entrepreneurial intention of entrepreneurs. Therefore, it improves the level of entrepreneurship and increases the output of entrepreneurship policy. Therefore, it can start with the establishment of entrepreneurial culture atmosphere to provide a good operating atmosphere for entrepreneurial policy. While emphasizing the impact of the external cultural environment on entrepreneurship, enterprises and entrepreneurs should also actively take measures to improve adaptability. Because the competitive environment is undergoing fundamental changes. Therefore, enterprises should refocus resources, fundamentally change their business methods, and create effective strategic changes [55].

Population. As for the effect of population factors on entrepreneurship policy, this study reveals two internal relations from interviews: First, the number, density, and mobility of the population are linked to the effectiveness of entrepreneurship policy through entrepreneurial opportunities and entrepreneurial intention. With a large population and a high density, the potential market demand of the region is large, so it is easy to identify and find entrepreneurial opportunities and influence the entrepreneurial intention of entrepreneurs. However, the high mobility of population indicates that there are few barriers to entry and exit of the market, which provides convenience for start-ups to enter the market. This is also in line with Fogel’s [56] principle of “reducing barriers”. As a result, the population size, density and mobility provide help for social entrepreneurship and indirectly affect the audience of entrepreneurship policy. Second, the quality of human resources affects the effectiveness of entrepreneurship policy by having the effect on the cost and output of start-ups. According to interviews, there are a large number of university students in Wuhan so that start-up enterprises can recruit high-quality employees at lower labor costs. Therefore, start-up enterprises can recruit high-quality employees at a lower labor cost and high-level talents easily. For example, “*A03 There are many colleges and college students, so we are easy to find high-level employees*.” It is of great importance to improve the productivity of start-ups. It can be seen that abundant human resources can affect the effect of entrepreneurship policy by reducing costs and increasing output.

## Conclusion

This study applies the method of grounded theory to conduct in-depth interviews with entrepreneurs in Wuhan. The interviewed entrepreneurs come from Wuhan University, Central China Normal University, Wuhan University of Technology, Wuhan Hongshan District Venture Center, Optics Valley Venture Coffee, and Qingshan Mass Entrepreneurship Center. The analysis found that policy formulation, science and technological support, legal guarantee, policy implementation, and social environment have significant effects on the effectiveness of entrepreneurship policy, and thus established the model of influencing factors of the effectiveness of entrepreneurship policy. Through comparison and analysis, it is found in this model that policy formulation, scientific and technological support, and legal guarantee are independent variables, policy implementation is a mediating variable, and social environment is a moderator variable. The above five categories have different ways of influencing the effectiveness of entrepreneurship policy.

### Theoretical contribution

The theoretical implications of this paper are mainly reflected in the following three aspects: First, this study further improves the theory of entrepreneurship management from the perspective of government public management. Most of the previous studies on entrepreneurship started from entrepreneurs to explore the interaction between entrepreneurs and entrepreneurial environment. This paper studies the influencing factors of the effectiveness of entrepreneurship policy from the perspective of governmental public management. On the one hand, this study enriches the connotation of entrepreneurial management theory and expands its theoretical extension. On the other hand, it promotes the integration of entrepreneurial management and public management research. Second, by clarifying the factors that affect the effectiveness of entrepreneurship policy, this study provides a theoretical basis for government scientific decision-making, and enhances the rationality and feasibility of the government to formulate entrepreneurship policy. Therefore, the theory of public decision in entrepreneurship policy is expanded. Third, this paper establishes a complex model that integrates many factors that affect the effectiveness of entrepreneurship policy. The model provides a theoretical basis for future research on the formulation, implementation and evaluation of entrepreneurship policy.

### Managerial implications

This paper also has managerial implications in management. First, the current economic development of world has entered the new normal, and it needs to shift from factor-driven and investment-driven to innovation-driven. It encourages mass innovation and entrepreneurship to create more jobs and stimulate the whole society’s potential for innovation and entrepreneurship. The research on the effectiveness of government entrepreneurship policy adapts to this practical need. Second, the current survival rate of small and medium-sized enterprises is not high enough because of the high risk of entrepreneurship. By studying and clarifying the factors that are favorable and unfavorable for entrepreneurial activities, it is helpful to small and medium-sized enterprises to grasp the policy dividend, seek benefits, avoid disadvantages, and promote the formation of an atmosphere of mass entrepreneurship and innovation. Third, compared to Europe and the United States and other developed countries, the entrepreneurship ability is relatively weak at present in China. The reason is largely due to imperfect entrepreneurship policy, and there are problems of insufficient and excessive policy support at the same time. The study on the effectiveness of entrepreneurship policy is able to effectively solve these problems and enhance social creativity and national innovation ability. Fourth, from the perspective of complex systems, the factors affecting the effectiveness of entrepreneurship policy involve interactions among multiple subsystems, such as policy, economics, finance, law, and society. The path of interaction between these subsystems is uncertain. The analysis framework provided in this paper, on the one hand, determines the content of the subsystems, on the other hand, it clarifies the relationship between these subsystems. In actual public governance, a system dynamics model can be established for simulation based on the framework provided in this paper, so as to provide help for predicting and evaluating the effectiveness of entrepreneurship policy.

### Limitations and future research

This paper has several limitations. First, this paper adopts the qualitative grounded theory method to study the problem, so it needs to further verify it. Second, the data only comes from Wuhan rather than other regions involved. There are diverse entrepreneurial policies and different economic, demographic, and industrial environment in different cities. The factors that influence the effectiveness of entrepreneurship may vary. The future research will strengthen the empirical quantitative research to verify the model of influencing factors of the effectiveness of entrepreneurship policy that is established in this paper and the relationship between all variables. Also, the future research is encouraged to extend the scope of data collected to the whole country in order to supplement and improve this model.
