# Correction: Antecedents of the effectiveness of entrepreneurship policy: An integrated framework

**DOI:** 10.1371/journal.pone.0346870

**Published:** 2026-04-08

**Authors:** Mingyang Zhang, Wei Zhang

An additional affiliation is missing for the first author. Mingyang Zhang is also affiliated with the School of Public Administration, Sichuan University, Chengdu, Sichuan, P.R. China.

## References

[1] Zhang M, Zhang W. Antecedents of the effectiveness of entrepreneurship policy: An integrated framework. PLoS One. 2026;21(2):e0247988. doi: 10.1371/journal.pone.0247988 41671276 PMC12893556

